# Multimorbidity of chronic diseases and health care utilization in general practice

**DOI:** 10.1186/1471-2296-15-61

**Published:** 2014-04-07

**Authors:** Sandra H van Oostrom, H Susan J Picavet, Simone R de Bruin, Irina Stirbu, Joke C Korevaar, Francois G Schellevis, Caroline A Baan

**Affiliations:** 1 Centre for Nutrition, Prevention and Health Services, National Institute for Public Health and the Environment, Bilthoven, the Netherlands; 2 Netherlands Institute for Health Services Research (NIVEL), Utrecht, the Netherlands; 3 Department of General Practice and Elderly Care Medicine/EMGO Institute for health and care research, VU University Medical Centre, Amsterdam, the Netherlands

**Keywords:** Multimorbidity, Chronic disease, Epidemiology, Health care utilisation

## Abstract

**Background:**

Multimorbidity is common among ageing populations and it affects the demand for health services. The objective of this study was to examine the relationship between multimorbidity (i.e. the number of diseases and specific combinations of diseases) and the use of general practice services in the Dutch population of 55 years and older.

**Methods:**

Data on diagnosed chronic diseases, contacts (including face-to-face consultations, phone contacts, and home visits), drug prescription rates, and referral rates to specialised care were derived from the Netherlands Information Network of General Practice (LINH), limited to patients whose data were available from 2006 to 2008 (N = 32,583). Multimorbidity was defined as having two or more out of 28 chronic diseases. Multilevel analyses adjusted for age, gender, and clustering of patients in general practices were used to assess the association between multimorbidity and service utilization in 2008.

**Results:**

Patients diagnosed with multiple chronic diseases had on average 18.3 contacts (95% CI 16.8 19.9) per year. This was significantly higher than patients with one chronic disease (11.7 contacts (10.8 12.6)) or without any (6.1 contacts (5.6 6.6)). A higher number of chronic diseases was associated with more contacts, more prescriptions, and more referrals to specialized care. However, the number of contacts per disease decreased with an increasing number of diseases; patients with a single disease had between 9 to 17 contacts a year and patients with five or more diseases had 5 or 6 contacts per disease per year. Contact rates for specific combinations of diseases were lower than what would be expected on the basis of contact rates of the separate diseases.

**Conclusion:**

Multimorbidity is associated with increased health care utilization in general practice, yet the increase declines per additional disease. Still, with the expected rise in multimorbidity in the coming decades more extensive health resources are required.

## Background

The presence of multiple coexisting chronic diseases in individuals and the expected rise in chronic diseases over the coming years are increasingly being recognized as major public health and health care challenges of modern societies [1-6]. Individuals with multiple conditions are presumed to have greater health needs, more risk of complications, and more difficulty to manage treatment regimens. At present the main health care model is disease-focused rather than person-focused. Therefore involvement of several different health care providers in managing multiple disorders is inevitable and often results in competing treatments, sub-optimal coordination and communication between care providers, or unnecessary replication of diagnostic tests or treatments [3,7,8]. Hence, the common belief is that persons with multiple diseases have high rates of health care utilization and this is confirmed by some international studies [4,9-14]. However, till now there is only limited information on health care utilization patterns related to multiple disorders in the Netherlands.

Primary care based registers represent a valuable source to study the relationship between multimorbidity and health care utilization. The general practitioner is usually the first health care professional confronted with patients’ health problems. Studies exploring care utilization in primary care registers showed that individuals with multiple chronic conditions had more contacts with general practice than those with single conditions [4,11-13]. An important question is whether such an increase in contacts is equal for each additional disease or whether the increase levels off from a certain number of diseases or for specific combinations of diseases. During a general practitioner visit several overlapping health conditions may be discussed. It is also possible that the increase in the number of contacts increases with each extra disease, due to competing treatment demands or polypharmacy [15]. More insight in the extent of the increase in contacts informs about the burden of multimorbid patients on health resources and may assist in planning and improving (the organization of) health care services.

The objective of this study was to examine the relationship between having multiple diseases and the number of contacts with general practice. We were specifically interested in the development of the number of contacts per additional disease and for specific combinations of chronic diseases.

## Methods

### Dataset

Data were derived from electronic medical records from general practices that participate in the Netherlands Information Network of General Practice (LINH) [16]. The network is a dynamic pool of practices, with yearly changes in composition. The dataset includes routinely recorded data on consultations, including medication prescriptions and referrals to medical specialists of all patients listed in the participating practices. Dutch inhabitants have a legal obligation to be registered with a general practice. Diagnoses are coded by the general practitioners using the ICPC-classification (International Classification of Primary Care) [17]. The effect of having multiple diseases on health care utilization was analysed using data from all patients of 55 years and older, since a substantial part (more than 10%) had multiple chronic diseases [18]. Given that diagnoses are recorded based on patient contacts, the number of patients with some specific chronic diseases is underestimated when medical records from one year are used [18]. Therefore, all patients of 55 years and older registered from 2006 to 2008 in a general practice within the LINH network were selected. This resulted in a study population of 32,583 patients in 36 general practices.

### Chronic disease and multimorbidity

Multimorbidity was defined as the co-occurrence of two or more of a list of 28 chronic diseases within one person [19]. We selected 28 highly prevalent, chronic and severe diseases (see Additional file 1 for the list of diseases and their corresponding ICPC codes) [20]. Health care utilization for specific comorbidities was explored for the following ten most prevalent chronic diseases: diabetes mellitus, coronary heart disease, osteoarthritis, chronic obstructive pulmonary disease (COPD), chronic back- or neck disorders, cancer, stroke, depression, heart failure, and anxiety disorders.

Episodes of care were constructed to determine whether or not a patient had a particular chronic disease. Episodes of care included all patients contacts and drug prescriptions pertaining to a specific health problem [21]. Thus two consultations for the same health problem are grouped into one episode of care [22,23]. Consider, for instance, a patient who visits the general practitioner with a chronic cough, and a few months later the same patient is diagnosed with COPD. Most likely, both diagnoses refer to the same health problem and to avoid double counting the two diagnoses were grouped into one episode of care named COPD. Another example is a patient with symptoms of breathlessness and a diagnosis of heart failure a few weeks later, these health problems were grouped into one episode of heart failure. The assumption was made that a chronic disease, once recorded, remains prevalent during follow-up years in the registration.

### Health care utilization

Health care utilization was defined as the number of contacts with general practice, the number of medication prescriptions, and the number of referrals in 2008. Data were recorded in the medical records as CTG-codes (declaration fee-for-service codes for each type of contact) as defined by the Dutch Care Authority (NZA). The following contact types where extracted and analyzed: face-to-face consultations, telephone contacts, home visits, diagnostics and minor surgery, and contacts for prescribing medication [24]. The total number of contacts was calculated as the sum of these five categories. Data about prescriptions according to the Anatomical Therapeutic Chemical classification system were available for 29 general practices, whereas data about referrals to specialized care were available for 26 general practices.

### Analyses

The total number of contacts in general practice according to sex and age was calculated for patients without a chronic disease, patients with one chronic disease, and patients with multimorbidity. Negative binomial regression analyses with a log-link function were used to determine the relationship between having multiple diseases and the number of contacts with general practice. Generalized estimated equations (GEE) with an exchangeable correlation structure were applied to account for clustering of patients in practices. All analyses were adjusted for sex and age. The total number of contacts was determined for the ten specific chronic diseases and the number of comorbid diseases.

To determine whether the increase in the number of contacts was equal for each extra disease, we calculated the number of contacts per disease. The number of contacts per disease were determined as the absolute number of contacts per disease (without paying attention to the number of total contacts for patients without any chronic disease) and as the number of additional contacts per disease (by subtracting the sex- and age-specific number of total contacts for patients without any chronic disease).

To study the number of contacts for specific combinations of chronic diseases, negative binomial regression models including an interaction between the diseases were used. All models were adjusted for sex, age, the number of other chronic diseases, and clustering of patients within general practices. All analyses were performed in SAS version 9.2 (SAS Institute, Cary, North Carolina, USA).

## Results

Over a fourth (26%) of the patients had multiple chronic diseases (15% had two diseases, 7% had three diseases, 3% had four diseases, and 1% had five or more diseases), 30% had one chronic disease, and 44% had no chronic disease.

### Multimorbidity and contact rates

Multimorbid patients had significantly more face-to-face consultations, telephone consultations, home visits, diagnostics or minor surgeries, and contacts for prescribing medications in general practice than patients with one disease or without any chronic disease (Table 1). The mean of the total number of contacts per year was 18.3 for patients with multiple diseases, 11.7 for those with a single chronic disease, and 6.1 for those without any chronic diseases. Patients with multimorbidity had a higher number of prescriptions and more referrals to specialized care (mean 27.5 prescriptions per year, 0.5 referrals per year) than those with one or without any chronic disease (mean 15.3 and 6.8 prescriptions per year, 0.3 and 0.2 referrals per year).

**Table 1:** Mean number of contacts with general practice in 2008 for persons without a chronic disease, with one chronic disease, and with multimorbidity

		**No chronic disease**	**One chronic disease**	**Multimorbidity**	**P-value for linear trend**
**Total contacts (sd)**^**1**^		**N = 14341**	**N = 9896**	**N = 8346**	
Sex	Male	5.3 (6.3)	11.2 (9.3)	19.0 (14.1)	
	Female	6.9 (7.4)	12.9 (10.2)	21.8 (15.5)	
Age	55 - 64 yrs	5.0 (5.8)	10.1 (8.3)	16.7 (12.8)	
	65 – 74 yrs	6.5 (6.8)	12.3 (9.5)	19.6 (14.9)	
	> = 75 yrs	10.2 (9.9)	15.8 (11.8)	24.0 (15.6)	
**Total contacts (95% CI)**^**2,3**^		6.1 (5.6 6.6)	11.7 (10.8 12.6)	18.3 (16.8 19.9)	<0.001
				
**Type of contacts (95% CI)**^**2**^					
Face-to-face consultations	GP^4^	2.3 (2.2 2.4)	3.6 (3.4 3.9)	4.8 (4.5 5.1)	<0.001
	PN^4^	0.1 (0.1 0.3)	0.5 (0.4 0.7)	0.7 (0.5 1.0)	<0.001
Phone consultations	GP	0.5 (0.4 0.6)	0.8 (0.7 1.0)	1.4 (1.2 1.8)	<0.001
	PN	0.1 (0.0 0.2)	0.2 (0.1 0.4)	0.4 (0.3 0.7)	<0.001
Home visits	GP	0.1 (0.1 0.1)	0.3 (0.3 0.4)	0.7 (0.6 0.9)	<0.001
	PN	0.0 (0.0 0.0)	0.0 (0.0 0.0)	0.1 (0.0 0.1)	<0.001
				
**Type of intervention (95% CI)**^**2**^					
Diagnostics and minor surgery		0.1 (0.1 0.2)	0.3 (0.2 0.3)	0.4 (0.4 0.5)	<0.001
Prescribing medication		2.8 (2.5 3.2)	5.8 (5.1 6.6)	9.3 (8.1 10.6)	<0.001
				
**Prescriptions**^**2,5**^		N = 11366	N = 7962	N = 7008	
Patiënts with prescriptions (N, %)		8005 (70.4%)	7396 (92.9%)	6897 (98.4%)	
Mean number of prescriptions		6.8 (6.1 7.7)	15.3 (13.8 16.9)	27.5 (24.9 30.5)	<0.001
				
**Referrals**^**2,.6**^		N = 10341	N = 7219	N = 6198	
Referred patiënts (N, %)		1549 (15.0%)	1822 (25.2%)	2230 (36.0%)	
Mean number of referrals		0.2 (0.2 0.2)	0.3 (0.3 0.3)	0.5 (0.4 0.5)	<0.001

^1^Crude means and sd’s.

^2^Adjusted for sex, age and clustering of patients in general practices.

^3^Total contacts consist of face-to-face consultations, telephone contacts, home visits, diagnostics and minor surgery, and contacts for prescribing medication.

^4^GP = general practitioner, PN = practice nurse.

^5^Among 29 general practices.

^6^Among 26 general practices.

**Figure 1:**
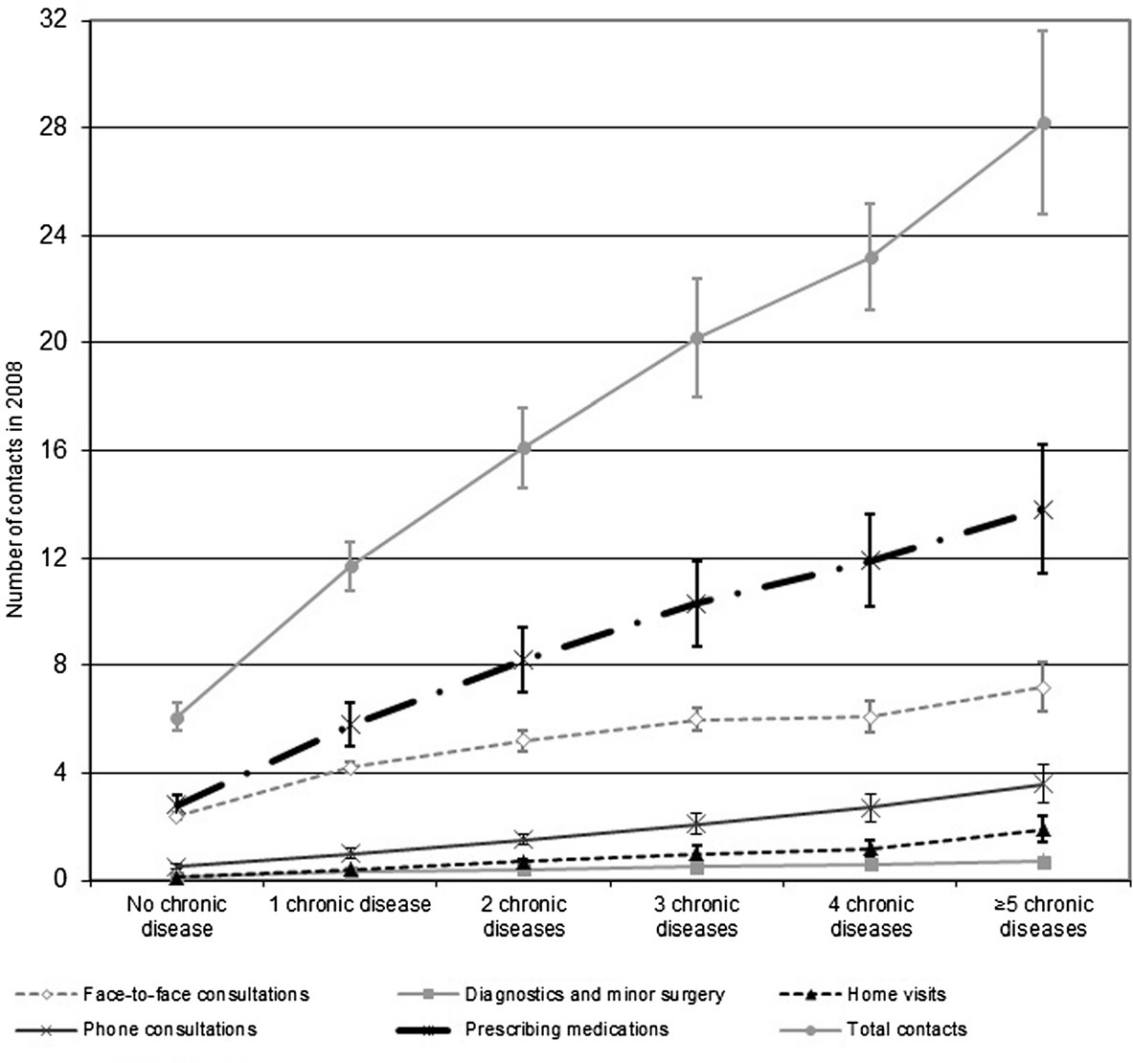
**Total number of contacts (and numbers of different types of contacts: face-to-face contacts, contacts for diagnostics and minor surgery, home visits, phone consultations, and contacts for prescribing medications) with general practice in 2008 for patients without a chronic disease and those with 1, 2, 3, 4 and 5 or more chronic diseases.** Means and 95% confidence intervals are presented, adjusted for sex, age, and clustering of patients within general practices.

The number of chronic diseases was linearly associated with the number of contacts for all types of contacts in general practice (Figure 1). For each of the ten specific chronic diseases we observed that the number of general practice contacts increased with the number of diagnosed comorbid diseases (Table 2). Patients with heart failure and comorbid diseases had most contacts with general practice.

**Table 2:** **Total number of contacts with general practice in 2008 for patients with ten prevalent chronic diseases grouped by their number of chronic diseases (out of 27 chronic diseases): means and 95% confidence intervals are presented, adjusted for sex, age, and clustering of patients within general practices**^**1**^

	**Number of patients**	**Single chronic disease**	**Number of additional chronic diseases**				**P-value for linear trend**
			**1**	**2**	**3**	**≥4**	
Diabetes	4806	14.4 (13.3 15.6)	18.5 (16.9 20.3)	23.3 (20.5 26.5)	26.6 (24.2 29.3)	32.3 (28.8 36.3)	<0.001
Coronary heart disease	3341	11.8 (10.7 13.0)	17.1 (15.4 18.9)	21.8 (19.8 24.1)	25.7 (23.3 28.3)	31.7 (27.7 36.2)	<0.001
Osteoarthritis	3394	10.8 (9.9 11.8)	16.2 (14.9 17.7)	20.7 (19.1 22.6)	23.4 (21.5 25.4)	28.4 (21.5 31.7)	<0.001
COPD	2466	13.6 (12.5 14.9)	17.2 (15.7 18.8)	22.4 (20.1 25.0)	25.3 (23.5 27.3)	32.1 (28.5 36.1)	<0.001
Chronic back- or neck disorder	2703	9.4 (8.7 10.3)	14.7 (13.4 16.2)	19.2 (17.3 21.4)	23.8 (21.3 26.6)	27.9 (24.3 31.9)	<0.001
Cancer	2269	11.5 (10.6 12.4)	16.8 (15.2 18.5)	21.4 (18.6 24.5)	23.1 (19.9 26.9)	32.0 (28.1 36.5)	<0.001
Stroke	1632	12.9 (11.8 14.2)	17.7 (16.1 19.4)	21.8 (19.5 24.4)	25.1 (22.6 28.0)	29.4 (26.4 32.8)	<0.001
Depression	1693	12.7 (11.5 14.0)	17.7 (15.8 19.9)	23.2 (20.2 26.6)	26.8 (24.3 29.5)	32.3 (28.4 36.8)	<0.001
Heart failure	1471	16.7 (14.8 18.8)	22.0 (19.9 24.3)	26.6 (24.3 29.2)	30.0 (27.3 32.9)	36.7 (33.2 40.5)	<0.001
Anxiety disorder	675	12.8 (10.9 15.0)	17.6 (15.2 20.4)	22.1 (19.1 25.5)	25.6 (21.8 30.0)		<0.001

^1^Means and 95% confidence intervals not shown when the number of patients with a chronic disease and a number of additional chronic diseases is lower than 50.

### Number of chronic diseases and contacts per disease

**Figure 2:**
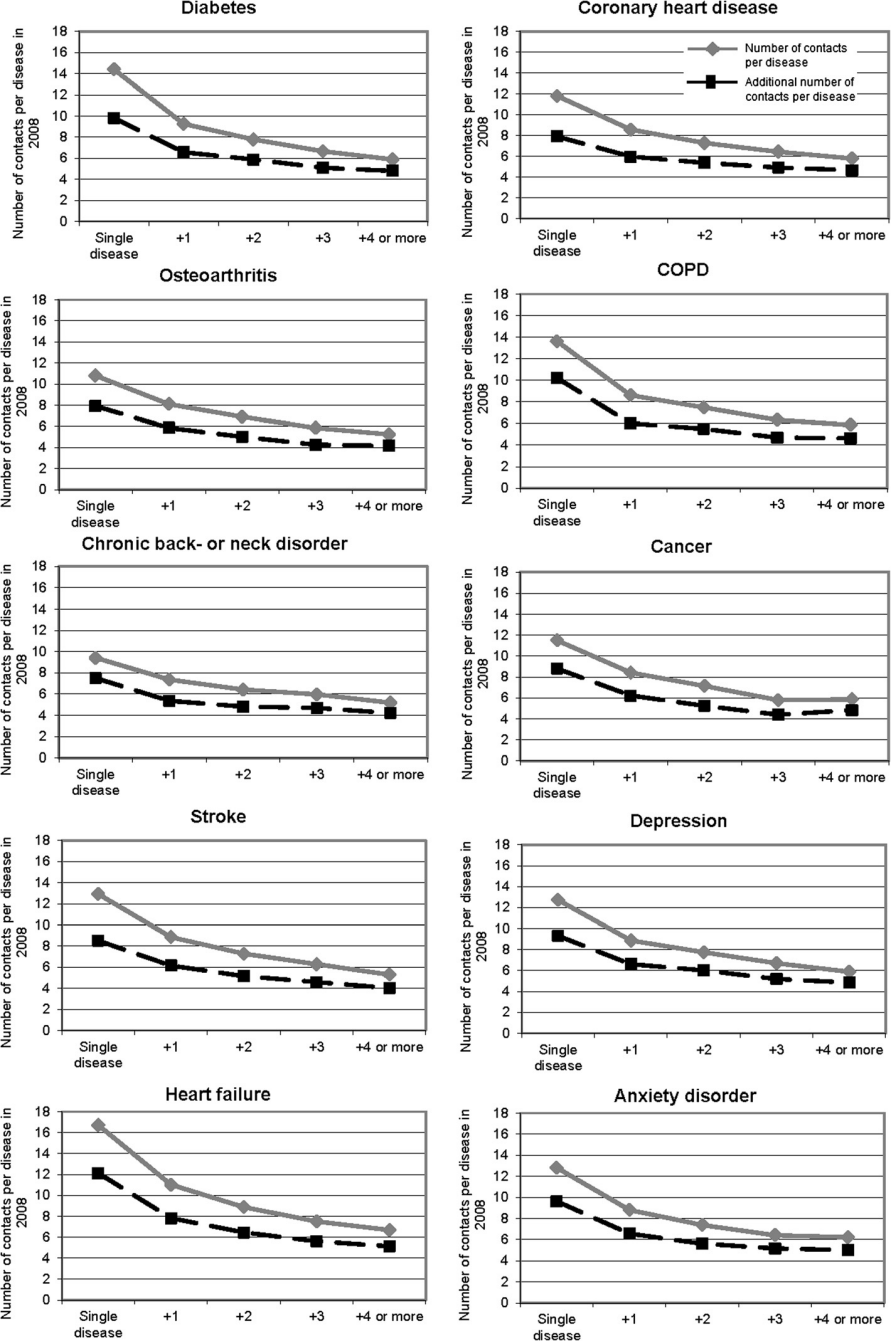
**Total number of contacts with general practice per disease in 2008 for patients with ten prevalent chronic diseases according to the number of diseases, adjusted for sex, age, and clustering of patients within general practices.** The grey solid line presents total number of contacts per disease, the black dotted line presents additional number of contacts per disease (minus the number of contacts for patients without chronic diseases).

We observed that the absolute number of contacts per disease decreased with each extra disease (Figure 2, grey solid lines). Patients with diabetes had 14.4 contacts per year and those with diabetes and four or more diseases had 5.9 contacts per disease per year. Contact rates of patients with a single disease varied for the ten diseases between 9 and 17 contacts per year. Patients with five or more diseases had 5 or 6 contacts per disease per year; this was very similar for each of the ten diseases. Additional contact rates per disease, shown by the black dotted lines, are lower than absolute contact rates per disease since the number of contacts for patients without any chronic disease was subtracted (Figure 2). Additional contact rates also showed a decline in the number of contacts per disease.

### Number of contacts for pairs of diseases

Almost all ratios (42 out of 45 disease pairs) showed a significantly lower number of contacts in general practice for those having specific combinations of two chronic diseases than what would be expected on the basis of contact frequencies of the chronic diseases individually (Table 3). The ratio is 0.78 for diabetes and coronary heart disease and the confidence interval indicates that the number of contacts per year is lower than expected on the basis of the number of yearly contacts of diabetes and coronary heart disease separately. There were no disease pairs where the number of contacts for the pair of diseases was higher than expected on the basis of the contact frequencies for the separate diseases.

**Table 3:** Ratios presenting the relative difference in the number of contacts in general practice for patients having specific combinations of diseases versus what would be expected on the basis of contact frequencies of those with either one of the diseases

	**Coronary heart disease**	**Osteoarthritis**	**COPD**	**Chronic back- or neck disorder**	**Cancer**	**Stroke**	**Depression**	**Heart failure**	**Anxiety disorder**
Diabetes	0.78 (0.73 0.82)	0.79 (0.75 0.84)	0.68 (0.63 0.73)	0.77 (0.71 0.83)	0.79 (0.71 0.87)	0.68 (0.63 0.72)	0.79 (0.71 0.88)	0.73 (0.68 0.78)	0.75 (0.67 0.84)
Coronary heart disease		0.81 (0.76 0.87)	0.75 (0.70 0.81)	0.88 (0.83 0.93)	0.78 (0.70 0.87)	0.73 (0.66 0.81)	0.76 (0.69 0.84)	0.69 (0.65 0.74)	0.77 (0.66 0.90)
Osteoarthritis			0.77 (0.72 0.83)	0.87 (0.80 0.95)	0.75 (0.67 0.85)	0.82 (0.75 0.90)	0.89 (0.81 0.98)	0.83 (0.76 0.90)	0.77 (0.68 0.87)
COPD				0.82 (0.76 0.89)	0.90 (0.80 1.01)	0.77 (0.70 0.85)	0.71 (0.63 0.80)	0.81 (0.75 0.86)	0.80 (0.70 0.91)
Chronic back- or neck disorder					0.79 (0.71 0.86)	0.82 (0.73 0.92)	0.91 (0.75 1.11)	0.78 (0.69 0.89)	0.94 (0.83 1.07)
Cancer						0.71 (0.64 0.79)	0.79 (0.69 0.91)	0.79 (0.72 0.87)	0.80 (0.69 0.94)
Stroke							0.73 (0.65 0.82)	0.84 (0.75 0.94)	0.73 (0.58 0.92)
Depression								0.71 (0.63 0.80)	0.69 (0.62 0.78)
Heart failure									0.78 (0.69 0.88)

Ratio and 95% confidence intervals are presented for patients with ten prevalent chronic diseases, adjusted for sex, age, number of other chronic diseases, and clustering of patients within general practices.

## Discussion and conclusions

The present study shows that patients with multiple chronic diseases had more contacts with general practice, more medication prescriptions, and more referrals to specialized care than patients with one or without any chronic disease. The number of contacts increased linearly with the number of chronic diseases for all types of contacts in general practice. However, the number of contacts per disease decreased with the number of diseases. In line with this finding, almost all patients with comorbid diseases had a lower observed number of contacts than would be expected on the basis of contact frequencies for each of the diseases separately.

Complex morbidity requires more diverse and intensive care [25], which likely explains the higher contact frequency among patients with multimorbidity. The finding of a lower number of contacts per disease is not so easy to interpret. A first explanation may be efficiency in treatment by the general practitioner; related health problems may be managed concurrently. Treatment or self-management strategies for diseases may overlap to a certain extent, and treatments may affect multiple diseases favourably [26]. Secondly, physicians and patients may also prioritize health problems, for instance to retain or reach an adequate level of patient’ well being or functioning [27,28]. Consequently, treatment for patients with multiple diseases may be suboptimal and chronic diseases may receive less attention than needed. Management of chronic diseases usually takes place in accordance with disease-specific guidelines, which pay only minor attention to treatment of patients with comorbidity, especially for diseases that are not related [29,30]. When comorbidity of diseases represents part of the same overall pathophysiologic risk profile or has overlapping treatment and self-management strategies (concordant diseases) [15,26], such as diabetes and coronary heart disease, a lower number of contacts might be expected. Management and treatment of concordant diseases generally affect the status of both diseases favourably [26]. However, for disease pairs that are not associated (discordant diseases) such as stroke and osteoarthritis the observed number of contacts was also lower than expected [15,26]. This corresponds with earlier findings that non diabetes-related comorbidity increases the health care demand as much as diabetes-related comorbidity [31]. A final explanation for a lower number of contacts per disease is that multimorbidity has a great impact on the balance of use of services between primary care and specialist physicians [9]. Two recent reviews concluded that having multiple diseases leads to a rise in specialized care, such as the utilization of specialist physician services, hospital admissions, and the number and length of hospital stays [1,14]. For a more comprehensive understanding of healthcare utilization for multimorbid patients, we should also look at the contact rates in specialised care. Specialists dominate the care of people with high burdens of morbidity because of the multiplicity of disease types. Therefore, substitution from primary care to specialised care may have occurred.

Obviously, the rise in the use of health care resources for patients with multiple diseases has consequences for the current and future burden of patients with multimorbidity in general practice, but the finding that per disease the number of contacts is lower may imply a more optimistic tendency. However, it should be noted that it is important to get insight into quality of care. Lower contact rates per disease may also indicate undertreatment. Quality and coordination of care for patients with multiple diseases is a concern since most treatments and guidelines are disease-specific [25,29,30]. Currently case-management programmes are being developed and evaluated worldwide for patients with multiple diseases, but it is still largely unknown what constitutes optimal care for multimorbid patients [10,32]. When quality of care is found to be low among patients with multiple diseases, a decreased number of contacts per disease is an undeserved trend. Underlying reasons for lower contact rates per disease must be explored in future studies.

Our findings are in line with other European studies exploring the relationship between the number of diseases and healthcare utilization in primary care [4,11-13]. German and English studies show that primary care utilization more than doubled for patients with multimorbidity (Germany mean 36.3 contacts per year, England 9.4 consultations per year) compared to those who are not multimorbid (Germany 15.9 contacts per year, England 3.8 consultations per year) [12,13]. As shown by the large differences in contact rates between these studies and with contact rates in our study, not just the number of diseases determines the number of contacts in primary care. The definition of contact rates and probably also accessibility of health resources affect the mean contact rates. For example, prescribing medication is included in our definition of a contact but not for the study in England [12]. This limits direct comparison of contact rates between countries. The reason that prescribing medications was included in our study is that it is actually a combination of prescribing medication and a telephone consultation, because questions about the medication or side-effects are very often discussed. Prescribing medication with or without telephone advice on medication issues cannot be distinguished in this general practice registration. Therefore, including the category of prescribing medication leads to an overestimation whereas excluding this category leads to an underestimation of general practitioner contacts with patients.

The German and English studies also confirm the linear increase of the number of chronic diseases with total contacts, a higher number of medication prescriptions, and a higher number of referrals for patients with multiple diseases [4,12,13]. The number of contacts per disease was not studied before. Our study is of interest since our findings showed lower contact rates per disease with an increased number of diseases and lower contact rates than what would be expected for specific combinations of diseases. For disease combinations costs in primary care were studied in another English study [33]. This study showed that the costs of treatment for most combinations of diseases did not differ from costs of two patients each with only one of the diseases. In total 12% of the combinations was cost-limiting, this was mainly observed among people over 60 years. Compared to our findings where almost all combinations of diseases showed lower contact rates than expected, cost-limiting conditions in the Brilleman study were less frequent. Moreover, about 7% of the combinations were cost-increasing and this was especially true for depression in combination with physical comorbidities (diabetes). It is not exactly clear why this differs from our results based on contact rates.

Main strengths of this study are the availability of data on diagnosed chronic diseases and the use of a large nationally representative sample of general practices. However, by using disease counts to define multimorbidity all chronic diseases contribute equally, independent of their severity or prognosis [34]. We noticed a large variation in contact rates between patients (shown by large standard deviations in Table 1), which may be explained by differences in the severity of health problems. It is likely that most severe health problems lead to the highest contact rates. For future research, it is interesting to study the variation in contact rates and identify the characteristics of patients with the highest care utilization. Moreover, disease counts do not account for differences in relationships between diseases. Concordant and discordant diseases are equally summed, while the impact on the patient may differ and be lesser or greater than the simple sum [35]. Although the use of disease counts has some limitations they are most widely used in multimorbidity research and they perform equally well compared to two other multimorbidity indices in determining the relationship with health care utilization [34]. Internationally there is a lot of variation in the number and type of chronic diseases that are considered in multimorbidity research and our selection also differed from others [6,36]. Generally speaking, the more chronic conditions are included the more patients with multimorbidity will be found. We presume that an important part of chronic morbidity is included in our selection of diseases. Furthermore, by using registration data from general practices the number of contacts for patients treated by specialists is not taken into account. The dataset only contained information about referrals to specialized care and no records of the number of contacts with specialists. As patients treated by specialist are mostly complex cases, we may assume that they usually have higher health care utilization.

Since the majority of older people have multiple diseases and their number is rising, it is important to get more insight in their health care utilization patterns. This study shows that the number of contacts in general practice increased linearly with the number of chronic diseases, thus multimorbid patients account for a high proportion of the healthcare workload. With the expected rise in multimorbidity in the coming decades, this requires more extensive health resources. Furthermore, the explanations for the decrease in contacts per disease should be explored. In case of undertreatment or low quality of care for patients with multiple diseases, advances may even lead to a further increased use of health resources in the future. In conclusion, health systems should be prepared for the future increase in utilisation of health services.

## Competing interests

The authors declare that they have no competing interests.

## Authors’ contributions

SHO and HSJP had the idea for the study. SHO analyzed the data and drafted the manuscript. HSJP, SRB, IS, JCK, FGS, and CAB critically revised the manuscript. All authors contributed to the interpretation of the results and approved the final manuscript. IS, JCK, and FGS contributed to the data collection.

## Pre-publication history

The pre-publication history for this paper can be accessed here:

http://www.biomedcentral.com/1471-2296/15/61/prepub

## Supplementary Material

Additional file 1 Selection of 28 chronic diseases with ICPC-1 codes. Click here for file
