# Supplementary material for: Multimorbidity of chronic diseases and health care utilization in general practice
Source: BMC Fam Pract. 2014 Apr 7;15:61. doi: 10.1186/1471-2296-15-61 (PMC4021063; doi:10.1186/1471-2296-15-61)
Supplement: Additional file 1 — Selection of 28 chronic diseases with ICPC-1 codes. [file 1471-2296-15-61-S1.docx]

| **Additional file 1**. Selection of 28 chronic diseases with ICPC-1 codes | |
| --- | --- |
| **Chronic disease** | **ICPC-1 code** |
| Tuberculosis | A70 |
| HIV/AIDS | B90 |
| Cancer | A79, B72, B73, D74, D75, D77, L71, N74, R84, R85, S77, T71, U75, U76, U77, W72, X75, X76, X77, Y77, Y78 |
| Chronic enteritis/ colitis ulcerosa | D94 |
| Visual disorder | F83, F84, F92, F93, F94 |
| Hearing disorder | H84, H85 |
| Congenital cardiovascular anomaly | K73 |
| Coronary heart disease | K74, K75, K76 |
| Heart failure | K77 |
| Stroke (including TIA) | K89, K90 |
| Chronic back or neck disorder | L83, L84, L85, L86 |
| Rheumatoid arthritis | L88 |
| Osteoarthritis | L89, L90, L91 |
| Osteoporosis | L95 |
| Congenital neurological anomaly | N85 |
| Multiple sclerosis | N86 |
| Parkinson’s disease | N87 |
| Epilepsy | N88 |
| Chronic alcohol abuse | P15 |
| Dementia | P70 |
| Schizophrenia | P72 |
| Anxiety disorder, neurosis, PTSS | P74, P79 |
| Depressive disorder | P76 |
| Mental retardation | P85 |
| Chronic obstructive pulmonary disease | R91, R95 |
| Asthma | R96 |
| Anorexia | T06 |
| Diabetes mellitus | T90 |
| The selection of chronic diseases is based on ‘Defining chronic conditions for primary care using ICPC-2: supplementary data'.[[18](#_ENREF_18)] ICPC-2 codes are encoded into ICPC-1 codes. | |
